# Endothelial cells by inactivation of VHL gene direct angiogenesis, not vasculogenesis via Twist1 accumulation associated with hemangioblastoma neovascularization

**DOI:** 10.1038/s41598-017-05833-9

**Published:** 2017-07-14

**Authors:** Ying Wang, Dan-Qi Chen, Ming-Yu Chen, Kai-Yuan Ji, De-Xuan Ma, Liang-Fu Zhou

**Affiliations:** Department of Neurosurgery, Huashan Hospital, Fudan University, Shanghai, 200040 China

## Abstract

Inactivation of the VHL tumour suppressor gene is a highly frequent genetic event in the carcinogenesis of central nervous system-(CNS) hemangioblastomas (HBs). The patterning of the similar embryonic vasculogenesis is an increasing concern in HB-neovascularization, and the classic vascular endothelial growth factor (VEGF)-mediated angiogenesis driven by VHL loss-of-function from human endothelium have been questioned. With this regard, we identify a distinct, VHL silencing-driven mechanism in which human vascular endothelial cells by means of increasing cell proliferation and decreasing cell apoptosis, is concomitant with facilitating accumulation of Twist1 protein in vascular endothelial cells *in vitro*. Importantly, this molecular mechanism is also pinpointed in CNS-HBs, and associated with the process of HB-neovascularization. In contrast with recent studies of HB-neovascularization, these modified cells did not endow with the typical features of vasculogenesis, indicating that this is a common angiogenesis implementing the formation of the vascular network. Taken together, these findings suggest that vasculogenesis and angiogenesis may constitute complementary mechanisms for HB-neovascularization, and could provide a rational recognition of single anti-angiogenic intervention including targeting to the Twist1 signalling for HBs.

## Introduction

Hemangioblastomas (HBs) are high-vascularized neoplasms commonly arising in the cerebellum or brainstem^1–4^. The cytological origin and process of neovascularization remains tremendously controversial. Etiologically, HBs are divided into two subtypes, the sporadic HBs (takes up 75%) and familial HBs (VHL disease) (takes up 25%). Histologic examination showed numerous small capillary channels and abundant vacuolated ‘stromal cells’ in sporadic HBs and VHL-HBs^2^. Currently, surgical complete resection is the only curative treatment for sporadic HBs; however, surgical treatment of brain-stem HBs is a great challenge to neurosurgeons because of the location of HBs. Moreover, as an inherited multisystem disorder, VHL-HBs are difficult to be cured. So a better understanding of HB biological mechanisms may further provide useful insights into its therapeutic strategies.

The cytological origin of HBs (including its neovascularization) and its evolutionary process remain controversial for nearly a century. The related studies are associated with the vascular progenitors including embryonic cells, reactive endothelial cells, stromal cells, and mast cells etc^5, 6^. In recent years, increasing evidence has showed that many mesodermal makers were expressed in both sporadic and inherited HBs^7, 8^, suggesting that HB-neovascularization is a similar embryologic vasculogenesis^9–12^. Then, as the classic hypothesis of HB-angiogenesis, the vascular endothelial growth factor (VEGF) secreted by stromal cells may bind to endothelial cells and trigger an intracellular signaling pathway in the endothelial cells that eventually results in cell proliferation and vascular formation^13^. In fact, this hypothesis originated from Folkman’s angiogenesis viewpoint (two cellular clonal origins); however, this conception also lacks the support of direct evidence in HBs. Therefore, whether this angiogenesis exists in HBs or what function is the angiogenesis in HBs, needs to be further elucidated.

We hypothesized that endothelial cells are the original cells of HB-neovascularization. Thus, human vascular endothelial cells could serve as an alternative cellular model for HBs. Although the exact etiology and the evolutionary process of HBs are unknown, the inactivation of the VHL tumor suppressor gene has been considered as the root cause of VHL-HBs^14, 15^. Epidemiological investigation revealed that most patients with VHL disease (60–80%) harbor HBs^16^. Moreover, loss of heterozygosity at the VHL gene locus had been also detected in the vascular component for all cases of sporadic HBs^17^. Furthermore, embryonic cell by acute inactivation of the VHL gene dramatically cause a great deal of placental vasculogenesis in the mice model^18, 19^. These evidences indicate that the inactivation of VHL gene is critical for vascular development in HBs, probably by mediating the process of HB-neovascularization.

Twist1 is an evolutionally conserved transcription factor, and is found in human normal mesodermal tissues^20^. The expression of Twist1represents a component of mesodermal programmingand control mesoderm specification and differentiations^21^. Increasing evidences have showed that up-regulation of Twsit1 expression is required for pathologic ocular angiogenesis^22^ and tumor angiogenesis^21, 23–25^. Thus, Twist1 can act as a marker for angiogenesis of specification and patterning of the mesoderm among evolutionarily distant organism^20^.

In the present study, we investigated the process of the vascular formation *in vitro*, focusing on the comparison with HB-neovascular characteristics.

## Results

### VHL Silencing Promoted HUVEC Proliferation and Decreased Apoptosis

**Figure 1:**
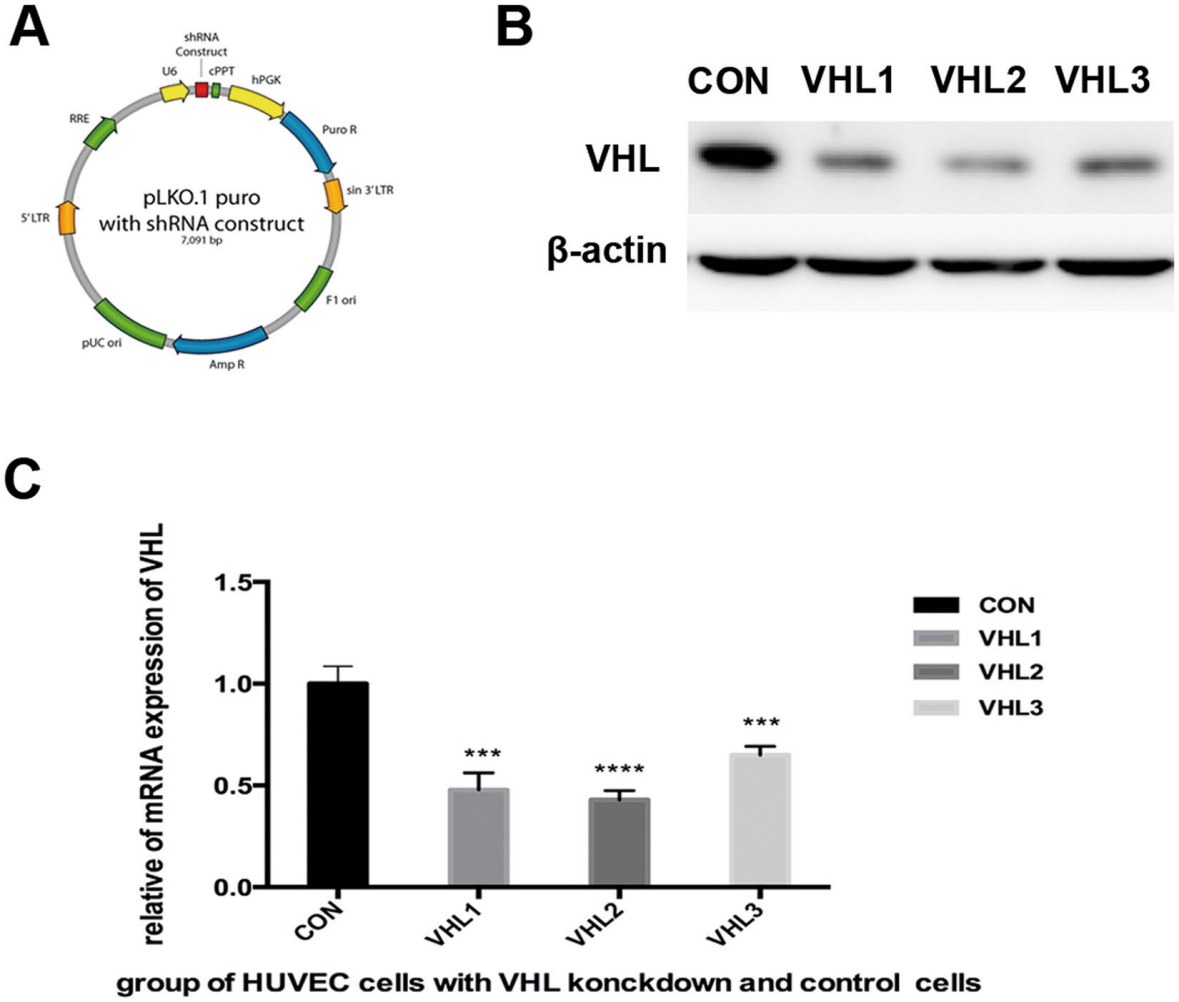
Knockdown of VHL gene expression in HUVEC cells by shRNA. The graph illustrated the schematic information of lentivirus vector pLKO.1 puro used for VHL shRNA construction (**A**). Western blot analysis (**B**) and qRT-PCR(**C**) analysis evaluated the knockdown efficiency of VHL gene in HUVEC cells 72 h after transduction. Statistical analysis was performed using unpaired Student’s t test, *** represent P < 0.001, **** represent P < 0.0001. The original figure of western blotting was shown in Supplementary Figure S1.

To ascertain the role of VHL gene silencing on the endothelial cells, we transfected retroviral vectors containing the sh-VHL into human umbilical vein endothelial cell (HUVEC). We assessed three consequences on the HUVEC. For each group, we generated cells with stable expression of shRNA targeting VHL and corresponding controls (pLKO.1) (Fig. 1A), and VHL expression levels were significantly reduced as confirmed by western blot (Fig. 1B). According to the qRT-PCR result, the knock-down efficiency of shVHL1–3 in HUVEC cells was 53%, 58% and 36%, respectively (Fig. 1C).

**Figure 2:**
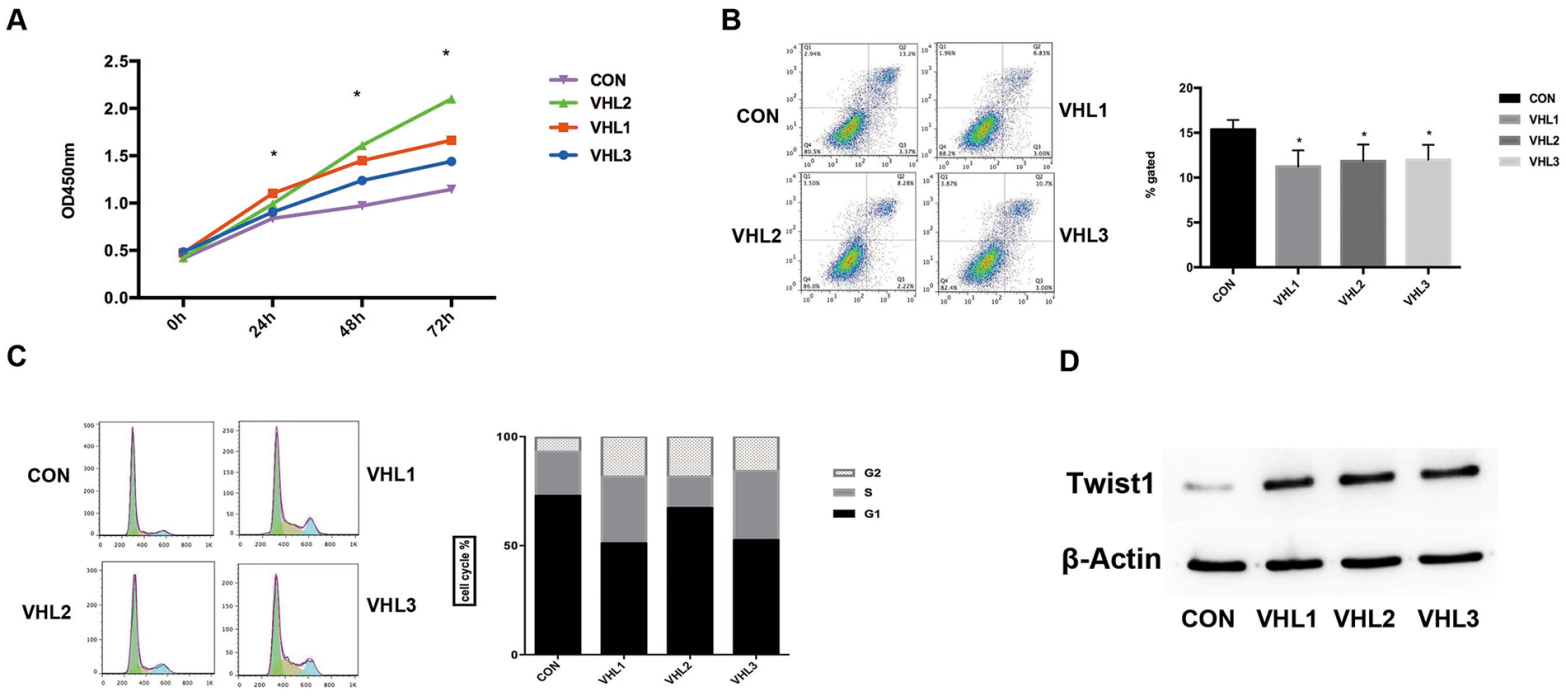
The effect of VHL gene knockdown on proliferation and apoptosis of HUVEC cells *in vitro*. Knockdown of VHL gene expression significantly promoted proliferation (**A**) and decreased apoptosis (**B**) of HUVEC cells *in vitro*. An increase in the percentage of cells in S or G2/M phase in sh-VHL groups was observed compared to the control group (P < 0.05) (**C**). Western blots results showed knockdown of VHL up-regulated Twist1 expression in HUVEC cells (**D**). All these data were representative of three independent experiments. Statistical analysis was performed using unpaired Student’s t test, * represent P < 0.05. The original figure of western blotting was shown in Supplementary Figure S2.

We first investigated the effect of VHL silencing on HUVEC viability using CCK8 assays by comparing viability of control and silencing cells over 3 consecutive days. The growth curve indicated that the cell proliferation was significantly faster in HUVEC (transfected with VHL shRNA) compared to those transfected with shRNA-Control (*P* < 0.05). We observed an increased viability in cells with VHL silencing relative to control cells (Fig. 2A).

Given that VHL repression increased cell viability, we next investigated whether this effect could be mediated through apoptosis and cell cycle control. We observed a decreased of apoptotic rates in HUVEC with VHL inactivation relative to control (*P* < 0.05) (Fig. 2B). This effect on apoptosis was consistent with an increase in the percentage of cells in S or G2/M phase in silencing versus control cells (Fig. 2C). Collectively, these results suggested that the modified cells by VHL inhibition elevated the cell viability by means of increasing cell proliferation and decreasing cell apoptosis, especially in accelerating S or G2/M phase within the cells cycles.

### VHL Silencing Promoted Angiogenesis in Matrigel-based Capillary Formation Assay and Spheroid Sprouting Assay

**Figure 3:**
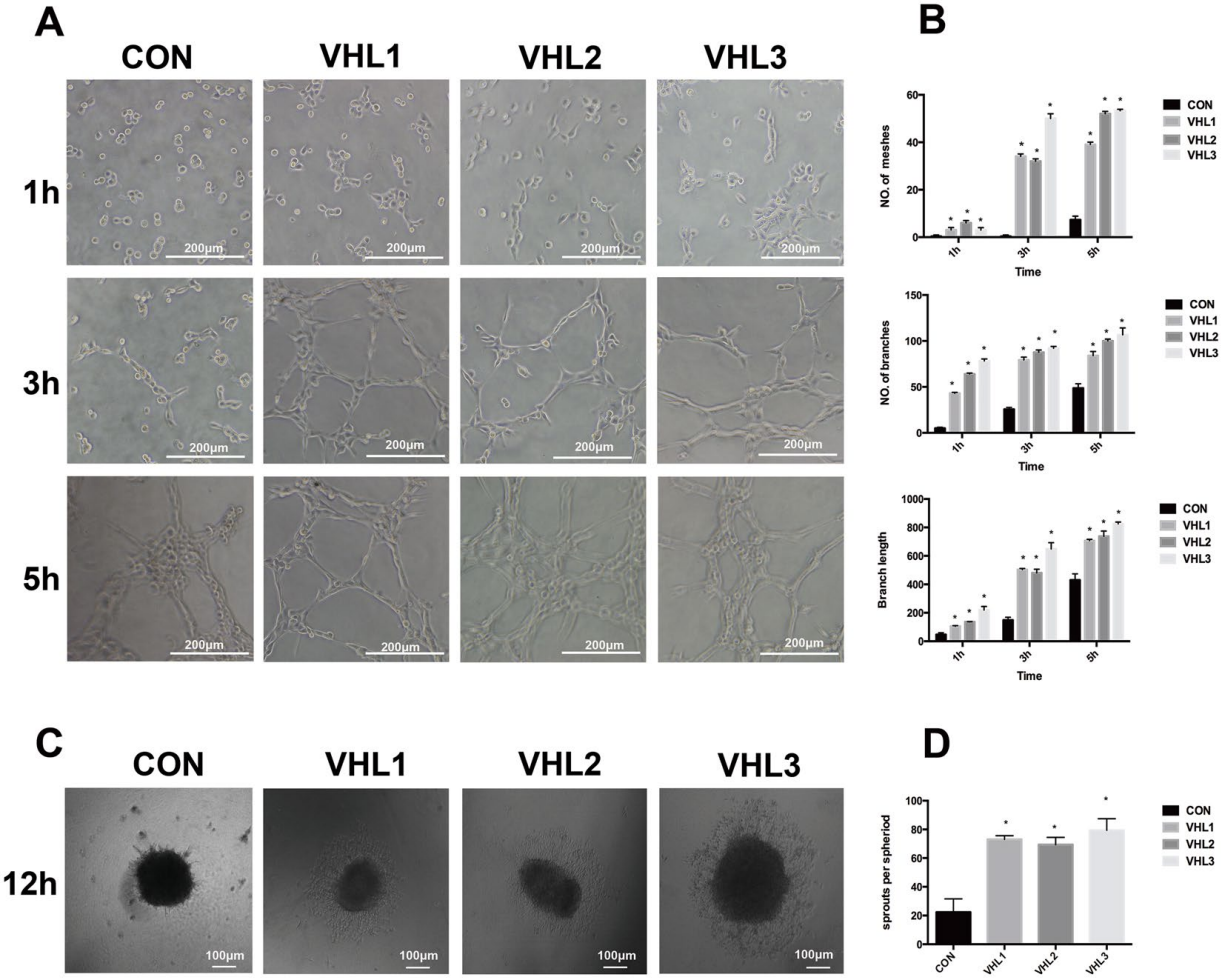
The effect of VHL gene knockdown on angiogenic ability of HUVEC cells in matrigel-based capillary formation assay and spheroid sprouting assay. (**A**) HUVEC cells from VHL knockdown groups exhibited a marked increase of capillary formation compared with control groups. At 1 hour, all cells did not form tube-structure. At 3 hours, VHL-silenced HUVEC exhibited the type of tube-structure while control cells did not. At 5 hours, all cells exhibited the type of tube-like structure with different morphologies, especially for typical features in VHL-silenced HUVEC cells (bar = 200 μm). The quantification data of tube formation assay was shown in (**B**). (**C**) Representative images of spout outgrowth after 12 hours in control and VHL-silenced HUVEC spheroids (bar = 100 μm). The length of the sprouts was measured and analyzed in (**D**), * represent P < 0.05.

To further investigate the potential role of VHL silencing in angiogenesis, we examined the effect of VHL shRNA on vascular endothelial tube formation and spheroid sprouting ability *in vitro*. In the matrigel-based capillary formation assay, we observed evident capillary structures as early as 3 hours after plating cells onto matrigel in HUVEC with VHL shRNAs. At the same time, the control cells did not form tube structures (Fig. 3A and B). In addition, we use the same method to establish two consequences (shVHL1 and 2) on human brain microvessel endothelial cell (HBMEC), VHL downregulation also increased the ability to form tube-like structures in HBMEC with VHL shRNAs. (Supplementary Figure S4)

In 3D spheroid sprouting assay, the HUVEC cells with VHL silencing also demonstrated increased sprout length of the spheroids by about 3-fold compared with control cells (Fig. 3C and D). VHL downregulation also increased sprout length of the spheroids in HBMEC (Supplementary Figure S4). These results indicated that VHL deficiency promoted the vessel-forming ability of human endothelial cells.

### Differentially Expressed Genes (DEGs), GO and KEGG Pathway Analysis

**Figure 4:**
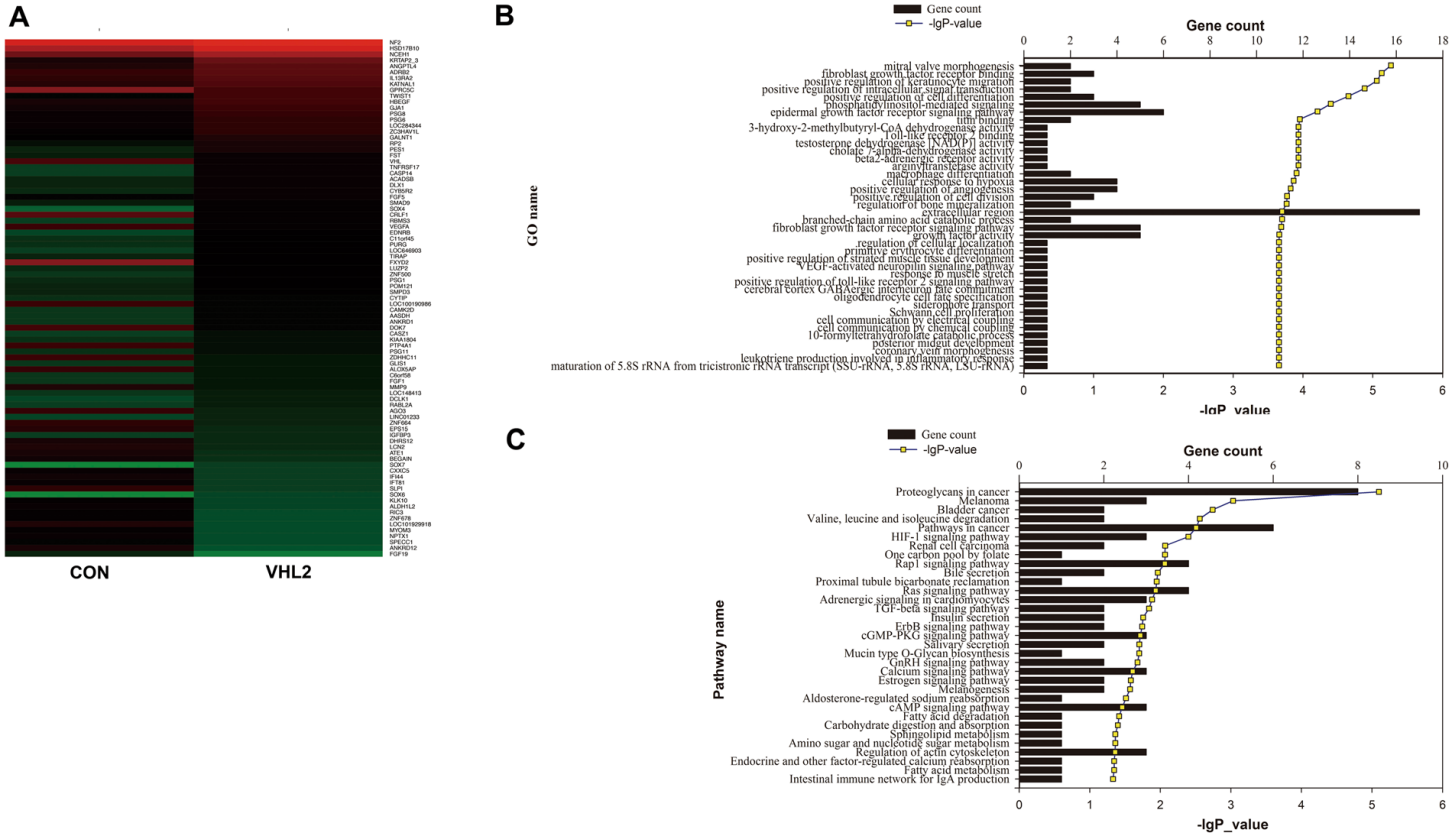
The difference of microarray gene expression profile between VHL-knockdown HUVEC cells and control group. The heat map (**A**) demonstrated the up-regulated and down-regulated genes in VHL-knockdown HUVEC cells compared to control group. The up-regulated genes were shown in red, while the down-regulated genes were shown in green. These up-regulated genes did not contain the specific mesodermal markers, such as SCl, brachyury and CD41. The Gene ontology (**B**) and KEGG pathway analysis (**C**) were used to demonstrate significantly changed pathways. The vertical axis is GO terms (up) and pathway category (down), and the horizontal axis is the gene count. P < 0.005 is used as a threshold to select significant GO categories, while P < 0.05 is used as thresholds to select significant KEGG pathway analysis.

Microarray was used to analyze the different gene between VHL-suppressed HUVEC and control cells. Following the statistical analysis, a total of 1325 transcripts were identified as differentially expressed (fold change > 2 or < 0.5, *P* < 0.05), in which a set of 642 genes were up-regulated and the remaining 683 genes were down-regulated. These up-regulated genes did not contain the specific mesodermal markers, such as SCl, brachyury and CD41, and were recently found in HB-neovascularization. Interestingly, Twist1, a marker for angiogenesis of specification and patterning of the evolutionally conserved transcription factor, was identified as the one of obvious up-regulated DEGs. The most valuable 86 DEGs (Fig. 4A) were selected to designate as GO categories with *P* < 0. 005. The GO categories for genes were associated with positive regulation of angiogenesis, positive regulation of cell division and so on (Fig. 4B). The significant KEGG pathways were designated as the 86 DEGs with P < 0.05. The significant pathways of DEGs after VHL inactivation were listed in Fig. 4C.

### Inhibition of VHL Resulted in Twist1 Overexpression

Among the DEGs, Twist1 was found as an important gene closely linked to VHL silencing, which has been reported as an important regulator of tumor neovascularization. To corroborate our finding of Twist1 expression associated with VHL inhibition in HUVEC, we analyzed the expression of Twist1 in HUVEC cells with VHL inactivation and control cells by western blot. Results showed that the expression level of Twist1 with VHL silencing up-regulated compare with the control cells (Fig. 2D). In addition, the VHL and Twist1 expression levels of HBMEC with VHL shRNAs were confirmed by western blot (Supplementary Figure S3).

### Expression of Twist1 in Primary Human HBs

**Figure 5:**
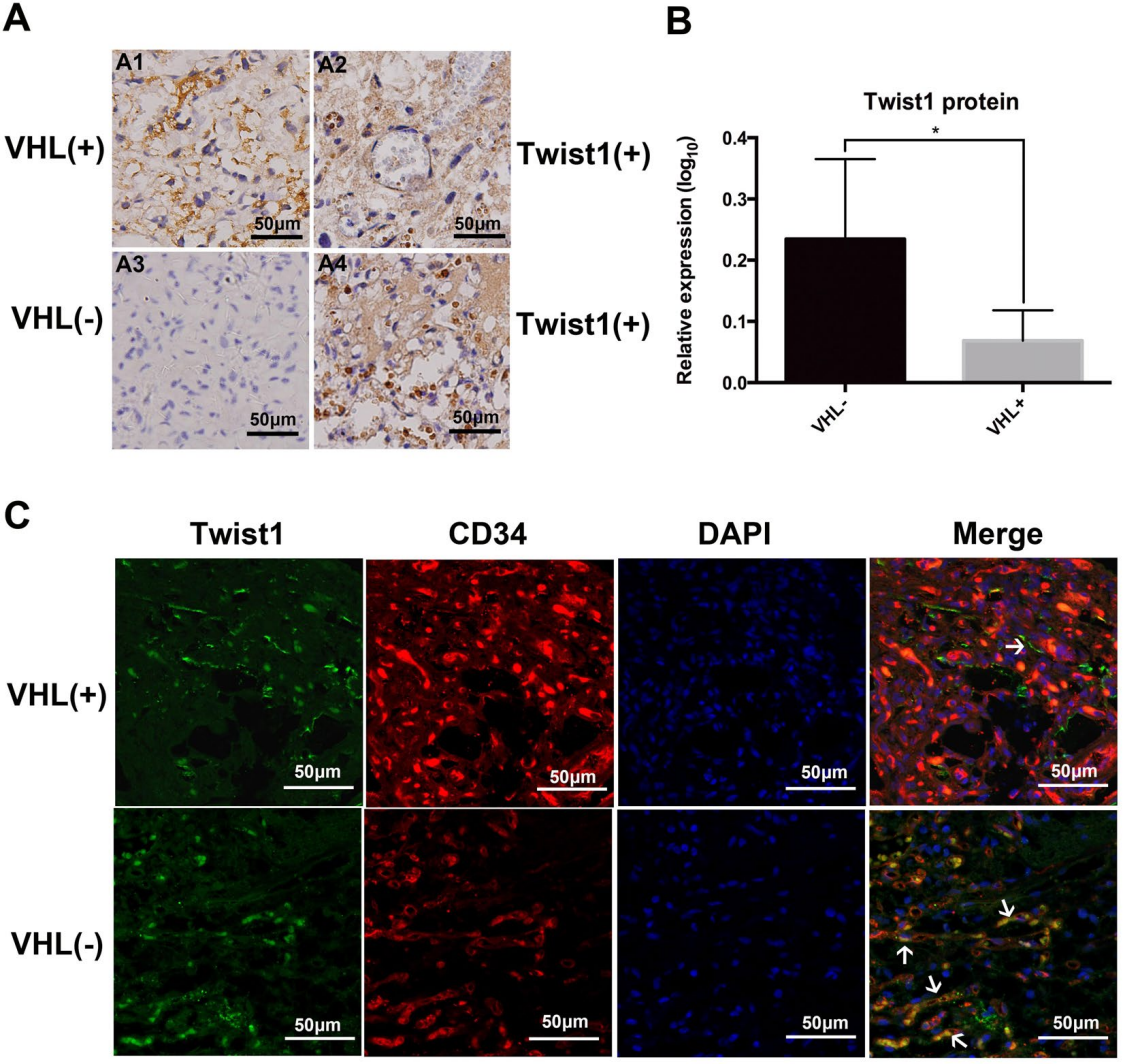
The correlation between VHL expression and Twist1 activation in HB tissues. (**A**) demonstrated that twist1 was over-expressed in VHL-negative HBs tissues (A3 and A4) compared with VHL-positive HBs tissues (A1 and A2) (Magnification × 400, bar = 50 μm). (**B**) Statistical analysis was performed using unpaired Student’s t test, *P < 0.05. IF results (**C**) revealed that Twist1 and CD34 were co-expressed in a small subset of cells in HBs tissues (Magnification × 400, bar = 50 μm). White arrows indicate the co-expressed cell.

To investigate the association between Twist1 expression and HBs neovascularization, we performed IHC and IF analysis. In this study, IHC showed that there was positive expression of Twist1 in both sporadic and inherited HBs of all tested 10 samples (Fig. 5A). Half of the ten samples were VHL positive expression, the other half was VHL negative expression. Twist1 was mainly expressed in the nuclei and cytoplasm while VHL was located in cytoplasm in tumor tissue. To further determine whether this loss of VHL function could result in the increase of Twist1 expression in HBs, we compared the two group samples. The result demonstrated that Twist1 expression was significant higher in the VHL negative expression tissues than in the VHL positive expression tissues (P < 0.05). Importantly, Twist1 immunoreactivity was observed in the CD34^+^ cells within human HBs by co-immunostaining. Combining with recent vasculogenesis of HB-neovascularization, this result indicated that these cells with CD34 expression harbored the angiogenic activity through Twist1 signaling, and the inactivation of VHL gene promotes the process.

## Discussion

Here we describe a distinct, VHL silencing-driven mechanism that provides recognition of vascular formation in human endothelial cells. These modified cells had enhanced potential of neovasculars by means of increasing cell proliferation and decreasing cell apoptosis, concomitant with facilitating accumulation of Twist1 protein *in vitro*. Importantly, this molecular mechanism is also identified in human HB tissues, and is associated with the process of HB-neovascularization. In contrast with recent studies of HB-neovascularization^9–12^, these modified cells did not endow with the typical features of vasculogenesis, indicative of that this is a common angiogenesis implementing the formation of the vascular network.

Tumors (solid neoplasms) are now recognized as organ-like tissues with extreme complexity^7–9, 12, 26^, and need to develop vascular network by supplying the tumor cells with vital nutrients and oxygen as well as disposing of metabolic wastes^27^. For the highly vascularized CNS-HBs, the exploration of the cytological initiation and its neovascular process not only has significance in their aetiology, but also provides potential insights into HBs’ anti-vascular treatment. However, this classic and predominant angiogenesis by sprouting and proliferation of endothelial cells from local vessels in CNS-HBs has been already questioned. With this regard, we hypothesize that human endothelial cells could act as a vascular origin cell of HB-neovascularization. By RNA interference for these cells in a manner analogous to the VHL-deficient vascular cells identified in areas of HBs, We found that they did not express a variety of specific phenotypic genes and the characteristic biological markers of HB-neovascularization, such as SCL, brachyury, Flk-1, SSEA1 and CD41^7–9, 12^, although these cells also showed elevated potential of neovascularization by means of increasing cell proliferation and decreasing cell apoptosis during the process of *in vitro* tube formation. Together with the observation that VHL-deficient mice embryonic cells obviously increased vasculogenesis^28, 29^, this data indicated that human endothelial cells might not dedifferentiate into and serve as the origin cells of HB-neovascularization.

Due to lack of cell lines and animal models of CNS-HBs, the research on process of initiation and development of HB-neovascularization become extremely difficult. It is widely accepted that mutation of the VHL gene is a frequent genetic event in the carcinogenesis of the highly vascularized HBs^16^, and the inactivation of the VHL function has been showed to be involved in the pathogenesis of both familial and most sporadic HBs^30, 31^. In this study, we show that reduction of VHL expression by specific shRNA in human endothelial cells leads to elevated angiogenic activity *in vitro*. Interestingly, the enhanced angiogenic response to accumulation of Twist1 protein, a novel VEGF/BFGF-independent VHL functions in the endothelium^32, 33^. Thus, partial loss of function of VHL in endothelium may be a contributing factor in tumor angiogenesis through a VEGF-independent mechanism.

Importantly, the Twist1-mediated signaling expression has been detected or confirmed in both sporadic and familial HBs, and has statistical significance concomitant with VHL loss-of-function. Of note, Twist1 and CD34 are co-expressed in HB vascular tissue. It is well known that CD34, widely found in HB vascular tissue, is a vascular progenitor cell/vascular cell marker, it commonly appear latter than some mesodermal marker, such as SCl,CD41,brancheyuryetc^7, 8, 12^ according to the committed differentiational process from embryonic cells to vascular cells. This result indicated that the putative vascular initiating cell-hemangioblasts have gained the potential of angiogenic ability through by activating Twist-mediated signaling during HB-neovascularization. Although our study do not support that the endothelium could serve as the original cell of HB-neovascularization, this high uniformity of Twist1 expression response to inactivation of the VHL gene, indicating that Twist1-mediated angiogenic signaling mechanism is conserved in the process of HB-neovascularization, at least in the process of the cell with CD34 expression. Combined with our previous studies of HB-vasculogenesis^9, 12^, a possible explanation may be that this angiogenic mechanism may play an accessorial role in development of HB-neovascularization, and might be responsible for the incorporation into foci of this vascular network, and sequential remodeling as well as expansion^34^.

Taken together, this research has uncovered the complexity of HB-neovascularization and provided a better understanding of HB-neovascularization, and will offer a novel attacking approach for anti-vascular therapy of VHL-HBs. Although the molecular mechanisms responsible for Twist1 in HB-neovascularization require further investigation, the major distinction with the previous conception is that both vasculogenesis and angiogenesis may constitute complementary mechanisms for HB-neovascularization. This result also implicated that anti-vascular therapeutic target for the Twist1 signaling, analogous to VEGF-mediated molecular mechanism^35^, maybe play a preventive role (incorporated into the preexisting vessels and remodeling) for VHL-HBs and could not inhibit HB growth because of involvement in angiogenic phase only.

## Methods

### Patients and Tissue processing

A total of ten patients, who underwent curative resection of CNS HBs at the Department of Neurosurgery, Huashan Hospital, Fudan University (Shanghai, China) between January 2011 and November 2012, were recruited in this study. The average age of the patients was 41.8 (17 to 68 years). Three were males and seven were females. Four patients were VHL disease cases while the other six patients were sporadic cases. The pathological diagnosis of HB was confirmed by 2 independent neuropathologists.

Formalin-fixed and paraffin-embedded tissues from these 10 HB patients were used for immunohistochemistry studies. This study was approved by the Research Ethics Committee of Huashan Hospital, Fudan University. Informed consent was obtained according to the committee’s regulations and the Declaration of Helsinki. The data did not contain any information that could lead to patient identification.

### Cell lines and Plasmids

The human umbilical vein endothelial cell (HUVEC) (Fudan IBS Cell Center, Shanghai), the human brain microvascular endothelial cell line (HBMEC) (iCell Bioscience, Shanghai) and human embryonic kidney cell line (HEK 293FT cell line) (ATCC, USA) were used in this study. These kinds of cells routinely maintained in Dulbecco’s modified Eagle’s medium (DMEM) (Gibco, BRL, Grand Island, USA) supplemented with 10% fetal bovine serum (FBS) (Gibco BRL), 100 unit/ml penicillin and 100 μg/ml streptomycin in a humidified 5% CO_2_ incubator at 37 °C.

Three VHL shRNA vectors (PLKO.1-shVHL1-3) of HUVEC cells and two VHL shRNA vectors (PLKO.1-shVHL1 and 2) of HBMEC cells were constructed according to the protocol of PLKO.1-puro vector (Addgene, Cambridge, MA). The forward oligonucleotide sequences of HUVEC cells and HBMEC cells were as follows:

ShVHL1- CCGGCCCTATTAGATACACTTCTTACTCGAGTAAGAAGTGTATCTAATAGGGTTTTTG,

ShVHL2CCGGGATCTGGAAGACCACCCAAATCTCGAGATTTGGGTGGTCTTCCAGATCTTTTTG,

ShVHL3-CCGGGCCTAGTCAAGCCTGGAATTCTCGAGAATTCTCAGGCTTGACTAGGCTTTTTG.

Transfer vector PLKO.1-shVHL1-3 or PLKO.1-shScramble, packing plasmid psPAX2 and envelope plasmid pMD2.G were co-transfected into in 293FT cells, by using the phosphate coprecipitation kit (Promega, Madison, WI, USA) according to manufacturer’s protocol and culture medium was replaced for 6 hours after transfection. The culture medium was replaced with DMEM medium (Gibco) containing 10% FBS (Gibco BRL). The virus containing media were collected at 48 hours after transfection. The cells were infected by lentiviral media for 72-hours. Subsequently, stable lines were selected by puromycin. These methods were performed in accordance with the approved guidelines and regulations from the Research Ethics Committee of Huashan Hospital, Fudan University.

### RNA isolation and quantitive reverse-transcription polymerase chain reaction (qRT-PCR)

Total RNA was extracted using Trizol reagent (Life Technologies, USA). The RNA was converted to cDNA using the reverse transcriptase kit (TaKaRa) according to the manufacture’s protocol. For qRT-PCR analysis, cDNA were amplified using SYBR Premix Ex Taq (TaKaRa). The PCR reactions were done in triplicates in following conditions: 95 °C/30 s, 35 cycles of 95 °C/5 s, 60 °C/15 s and 72 °C/10 s using the CFX96 Real-Time instrument, and the primers used for quantitative PCR were as follows: VHL-CTCAGCCCTACCCGATCTTAC/ ACATTGAGGGATGGCACAAAC, β-actin-GGCTGTATTCCCCTCCATCG/CCAGTTGGTAACAATGCCATGT. The relative expression was calculated using the comparative Ct method and was normalized by endogenous human β-actin for each sample. These methods were performed in accordance with the approved guidelines and regulations from the Research Ethics Committee of Huashan Hospital, Fudan University.

### Western blot analysis

The cultured cells were lysed in 2 × SDS lysis buffer and heated at 100 °C for 15 minutes. Proteins were loaded, separated in 12% SDS-PAGE gels and transferred to PVDF membrane soaked with absolute methanol. The membrane was blocked with 5% bovine serum albumin (BSA) for 30 min at room temperature and then incubated overnight at 4 °C with VHL antibody (Cell Signaling,1:500 for HUVEC and Abcam,1:200 for b. End3 cell line), Twist1 antibody (Santa Cruz,1: 100). After several washes with PBST, the membranes were incubated with HRP-conjugated goat anti-rabbit secondary antibody and anti-β-actin antibody at room temperature for 2 hours. After final washing with PBST, the signal was detected by chemiluminescence substrate (Millipore). These methods were performed in accordance with the approved guidelines and regulations from the Research Ethics Committee of Huashan Hospital, Fudan University.

### Cell viability, cell cycle and apoptosis assay

Cell viability was measured by the CCK8 assay (cell counting kit-8, Molecular Technologies, Shanghai, China). Following transfection for 72 hours, the cells were seeded into 96-well plate for an additional 3 days. 10 μl CCK8 solution was added into each well. Then the cells were incubated for 1 hour at 37 °C. Absorbance was measured at 450 nm on microplate reader (Bio-Rad Laboratories, USA).

The cell cycle analysis was examined by flow cytometry (FCM). The cells were harvested and fixed in ice-cold 70% ethanol at −20 °C overnight. The fixed cells were washed with ice-cold PBS and re-suspended in propidium iodide (PI) staining solution (Nexcelom). The stained cells were incubated at 37 °C incubator for 40 min in the dark. The cells were centrifuged at 1,200 r.p.m for 8 minutes. And cell cycle analysis was carried out in a FACS Cater-plus flow cytometer (Becton-Dickinson, USA)

Cell apoptosis was analyzed using the Annexing V/Propidium Iodide apoptosis kit (BD Biosciences Pharmingen, USA). The cells were re-suspended with 100 µl binding buffer and stained with1.5 µl Annexin V and 3 µl PI. Finally, the mixture was incubated for 10 min at room temperature in dark place and analyzed by FACS Cater-plus flow cytometer (Becton-Dickinson, USA). These methods were performed in accordance with the approved guidelines and regulations from the Research Ethics Committee of Huashan Hospital, Fudan University.

### Matrigel-based capillary formation assay

Matrigel solution (Abcam, USA) was thawed at 4 °C and quickly added to each well of a 96-well plate. The plate was incubated at 37 °C to allow the matrix solution to solidify. HUVEC cells with VHL shRNAs and control cells(5 × 10^3^ per well) in serum-free medium were seeded on to the gel and cultured at 37 °C for 3 and 5 hours. The formation of capillary-like structures was observed under an inverted light microscope. The number of the formed tubes, which represent the degree of angiogenesis *in vitro*, were scanned and quantitated in five low power fields. The matrigel-based capillary formation method of HBMEC was shown in supplementary method. These methods were performed in accordance with the approved guidelines and regulations from the Research Ethics Committee of Huashan Hospital, Fudan University.

### Spheroid sprouting assay

In order to generate the cell spheroids, a specific number of endothelial cells were suspended in the culture medium and seeded in the 3D round-bottom 96-well plates (S-Bio, Japan). In this condition, these kinds of cells contributed to the formation of the cell spheroids. The spheroids were harvested within 24 hours and embedded in matrigel in the 15-well plate (Ibidi, Germany). Then the plate was cultured at 37 °C in 5% CO_2_at 100% humidity. After 12 hours or 24 hours, images were taken and the cumulative sprout length was measured for per well. Experiments were repeated at least three times. These methods were performed in accordance with the approved guidelines and regulations from the Research Ethics Committee of Huashan Hospital, Fudan University.

### Microarray hybridization and data analysis

The shVHL2 group and control group of HUVEC cells were used to investigate the differential expression genes of VHL silencing. Total RNA was isolated using the Trizol Reagent (Life technologies, US). Qualified total RNA was further purified by RNeasy mini kit (QIAGEN, Germany) and RNase-Free DNase Set (QIAGEN, Germany). Microarray analysis was performed at ShanghaiBio Corporation (SHC, China) using Agilent Whole Human Genome Microarrays 4 × 44k (Agilent, USA). Total RNA was amplified and labeled by Low Input Quick Amp Labeling Kit, One-Color (Agilent technologies, US), following the manufacturer’s instructions. Labeled cRNA were purified by RNeasy mini kit (QIAGEN, Germany). Each slide was hybridized with 1.65 μg Cy3-labeled cRNA using Gene Expression Hybridization Kit (Agilent technologies, US) in Hybridization Oven (Agilent technologies, US), according to the manufacturer’s instructions. After 17 hours hybridization, slides were washed in staining dishes (Thermo Shandon, US) with Gene Expression Wash Buffer Kit (Agilent technologies, US), followed the manufacturer’s instructions. Slides were scanned by Agilent Microarray Scanner (Agilent technologies, US) with default settings, Dye channel: Green, Scan resolution = 5 μm, PMT 100%, 10%, 16 bit. Data were extracted with Feature Extraction software 10.7 (Agilent technologies, US). Raw data were normalized by Quantile algorithm, GeneSpring Software 12.6.1 (Agilent technologies, US).

Differentially Expressed Genes (DEGs) data were considered significant if both the P value < 0.05, fold change (FC) <= 0.5 or >= 2 after normalization using Gene Spring Software 12.6.1. The Gene Ontology (GO) annotations of the DEGs were downloaded from the GO project (http://www.geneontology.org) and NCBI (http://www.ncbi.nlm.nih.gov). The pathway analysis was obtained from the Kyoto Encyclopedia of Genes and Genomes (KEGG) database (http:// www.genome.jp/kegg). A Fisher exact test was used to find significant enrichment for pathways. GO categories and Pathway categories with a P value < 0.01 were reported. These methods were performed in accordance with the approved guidelines and regulations from the Research Ethics Committee of Huashan Hospital, Fudan University.

### Immunohistochemistry (IHC)

Tissue sections were deparaffinized twice by xylene and then hydrated. Hydrogen peroxide (0.6%) was used to eliminate endogenous peroxidase activity. The sections were blocked with goat serum in Tris-buffered saline for 30 min. Sections were then incubated with anti-VHL antibody (Abcam, 1:100) and anti-Twist1 antibody (Abcam, 1:500) overnight at 4 °C. Secondary antibody was then applied and incubated at 37 °C for 1 hour. Sections were developed with diaminobenzidine and stopped with water. Photographs of representative fields were captured using a Leica CCD camera DFC420 connected to a Leica DMIRE2 microscope (Leica Microsystems Imaging Solutions, Cambridge, UK). Quantification of immunoreactivity was performed on digitally captured color images saved as TIFF files and analyzed using Image-Pro plus 6.0 (Media Cybernetics, Rockville, Maryland, USA). These methods were performed in accordance with the approved guidelines and regulations from the Research Ethics Committee of Huashan Hospital, Fudan University.

### Immunofluorescence (IF)

For staining of paraffin sections, HBs tissue was sectioned at a thickness of 5 um. Sections were deparaffinised, rehydrated and stained. The primary antibodies were used: rabbit anti-Twist1 (Abcam, 1:500), mouse anti-CD34 (Abcam, 1:1000). Primary antibodies were visualized by secondary antibodies conjugated to Alexa Fluor 488,546(Invitrogen, 1:10000). After immunolabeling, cells were washed, stained with DAPI for DNA stain (Sigma), and viewed with confocal scanning microscope (Leica, Germany). These methods were performed in accordance with the approved guidelines and regulations from the Research Ethics Committee of Huashan Hospital, Fudan University.

### Statistical analysis

Statistical analysis was performed with Graphpad Prism. The difference between groups was assessed using a Student t test when comparing only two groups or one-way analysis of variance when comparing more than two groups. P < 0.05 was considered statistically significant.

## Electronic supplementary material

Supplementary Information
