# Supplementary material for: Endothelial cells by inactivation of VHL gene direct angiogenesis, not vasculogenesis via Twist1 accumulation associated with hemangioblastoma neovascularization
Source: Sci Rep. 2017 Jul 14;7:5463. doi: 10.1038/s41598-017-05833-9 (PMC5511164; doi:10.1038/s41598-017-05833-9)
Supplement: Supplementary file 1 — Supplementary Information [file 41598_2017_5833_MOESM1_ESM.pdf]

Supplementary information

Endothelial cells by inactivation of VHL gene direct angiogenesis, not vasculogenesis via Twist1 accumulation associated with hemangioblastoma neovascularization

Ying Wang[1], Dan-Qi Chen[1], Ming-Yu Chen[1], Kai-Yuan Ji[1], De-Xuan Ma[1, *]and Liang-Fu Zhou[1, *]

[1]Department of Neurosurgery, Huashan Hospital, Fudan University, Shanghai, 200040, China.

Correspondence and requests for materials should be addressed to D.-x.M (email:madexuan03@126.com) or L.-f.Z. (email: lfzhouc@126.com)

Supplementary Methods

Matrigel-based capillary formation assay for HBMEC

Matrigel solution (ibidi, Germany) was thawed at 4 ℃ and quickly added to each well of a 15-well plate. The plate was incubated at 37 ℃ to allow the matrix solution to solidify. HBMEC cells (2×10^4per well) in serum-free medium were seeded on to the gel and cultured at 37℃ for 12 hours. The formation of capillary-like structures was observed under an inverted light microscope. The number of the formed tubes, which represent the degree of angiogenesis in vitro, were scanned and quantitated in five low power fields .

RNA isolation and quantitive reverse-transcription polymerase chain reaction (qRT-PCR)

The shVHL1group and control group of HBMEC cells were used to investigate the relative mRNA expression of some specific mesodermal markers. The method of RNA isolation and qRT-PCR was the same in the manuscript. The primers used for quantitative PCR were as follows:

Branchyury-ACGACAACGGCCACATTATTC/CCTCGGCATATTTCTCGCTATCT

FLK1-GGCCCAATAATCAGAGTGGCA/CCAGTGTCATTTCCGATCACTTT

CD41-GATGAGACCCGAAATGTAGGC/GTCTTTTCTAGGACGTTCCAGTG

Supplementary Figures

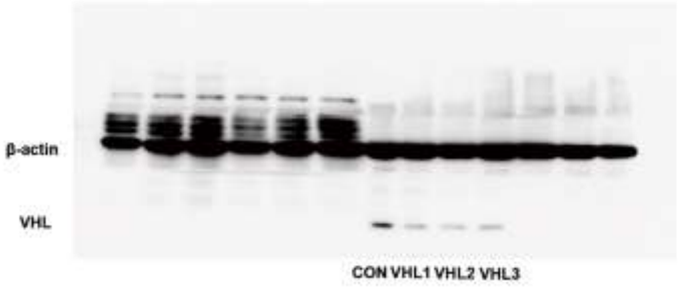

Supplementary Figure S1.The original figure of western blotting in figure 1. (B)

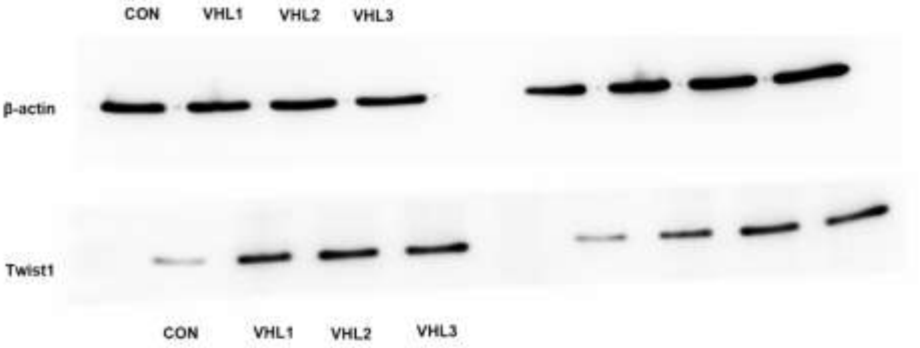

Supplementary Figure S2.The original figure of western blotting in figure 2. (D)

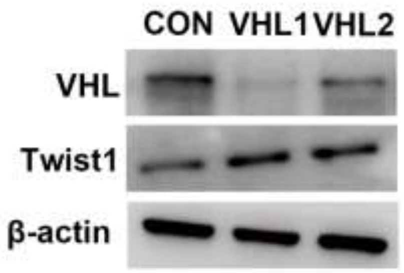

Supplementary Figure S3. Inhibition of VHL resulted in Twist1 overexpression in HBMEC by western blot.

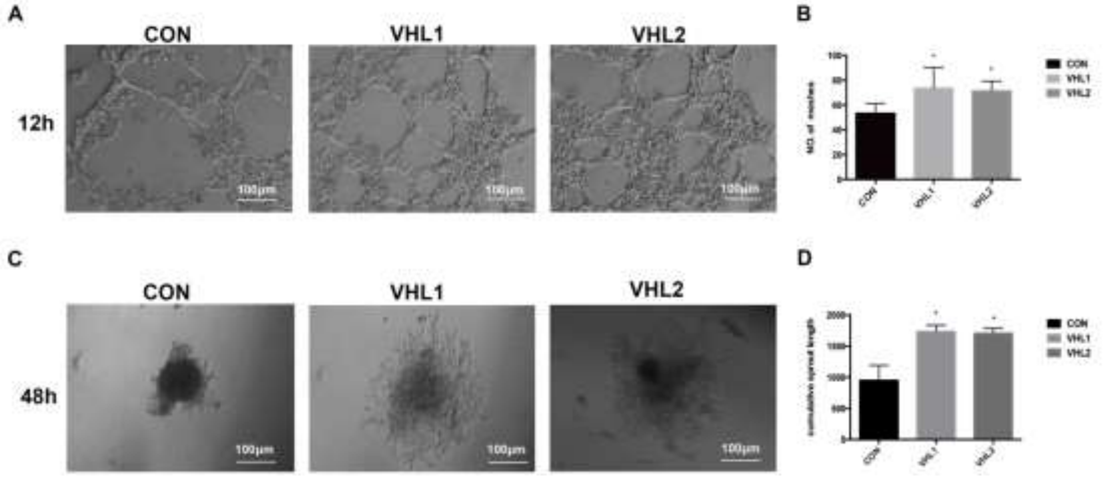

Supplementary Figure S4. VHL silencing promoted angiogenesis in matrigel-based capillary formation assay and sprouting angiogenesis assay in HBMEC.

HBMEC cells with VHL knockdown groups exhibited a marked increase of capillary formation compared to control groups. At 12 hours, all cells exhibited the type of tube-like structure with different morphologies (A), especially for typical features in VHL-suppressed cells (bar=100μm). The quantification data of tube formation assay was shown in (B). (C) Representative images of spout

outgrowth after 48 hours for control cells and VHL targeted shRNA treated HBMEC cells. (bar=100μm). The length (μm) of the sprouts was measured and analyzedin (D), * represent P<0.05.

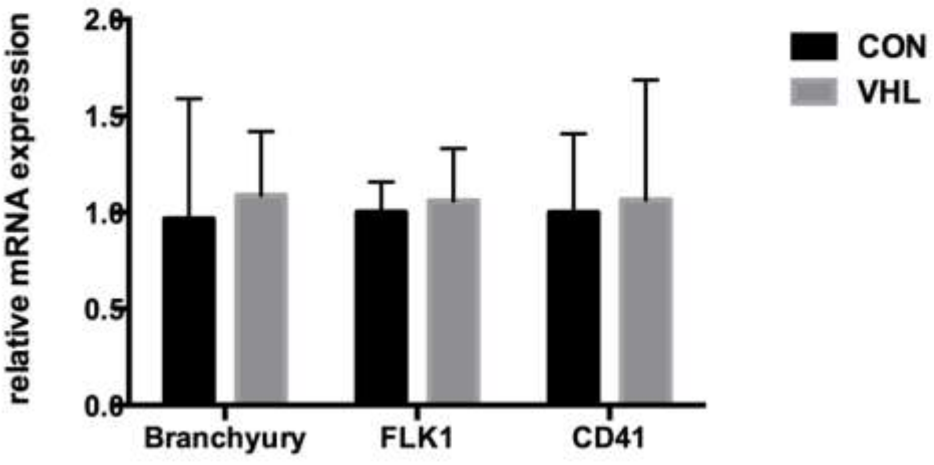

Supplementary Figure S5. Inhibition of VHL did not result in the specific mesodermal markers upregulation in HBMEC by qRT-PCR

The data demonstrated that control group and VHL down-regulated group of HBMEC was different, but the difference has no statistical significance. This data supported the above-mentioned result, Statistical analysis was performed using unpaired Student's t test.
